# Wellbeing-responsive community: a growth target for intentional mental health promotion

**DOI:** 10.3389/fpubh.2023.1271954

**Published:** 2023-12-13

**Authors:** Ivan J. Raymond, Karena J. Burke, Kylie J. Agnew, David M. Kelly

**Affiliations:** ^1^LBI Foundation, Adelaide, SA, Australia; ^2^College of Psychology School of Health, Medical and Applied Sciences, Appleton Institute, Central Queensland University, Adelaide, SA, Australia

**Keywords:** community wellbeing, mental health, intentional practice, community capacity building, health promotion

## Abstract

With mental illness remaining a significant burden of disease, there is an ongoing need for community-based health promotion, prevention, and responses (or “mental health promotion activities”). The health promotion, community development, and positive psychology literature identifies significant heterogeneity in the design and delivery of these activities. This variability spans: (1) individual vs. group outcomes, (2) psychological vs. sociological determinants of change, (3) promoting wellbeing vs. reducing mental health symptoms, and (4) the degree activities are contextualized vs. standardized in design and delivery. Mental health promotion activities do not easily accomplish this level of complexity within design and implementation. This has led to the emergence of the complexity-informed health promotion literature and the need for innovative tools, methods, and theories to drive this endeavor. This article directly responds to this call. It introduces “wellbeing-responsive community”: a vision and outcome hierarchy (or growth target) for intentionally delivered mental health promotion. The construct enables the design and implementation of interventions that intentionally respond to complexity and contextualization through the drivers of co-creation, intentionality, and local empowerment. It represents a community (support team, programme, agency, network, school, or region) that has the shared language, knowledge, methods, and skills to work together in shared intent. In other words, to integrate best-practice science with their local knowledge systems and existing strengths, and intentionally co-create and deliver contextualized wellbeing solutions at both the individual and community levels that span the “system” (e.g., whole-of-community) to the “moment” (e.g., intentional support and care). Co-creation, as applied through a transdisciplinary lens, is emerging as an evidence-based method to respond to complexity. This article describes the rationale and evidence underpinning the conceptualization of a wellbeing-responsive community through the integration of three key disciplines: (1) positive psychology, (2) ecological or systems approaches, and (3) intentional practice (implementation science). A definitional, contextual, and applied overview of the wellbeing-responsive community is provided, including a hierarchy of outcomes and associated definitions. Its purported application across education, mental health, community service, and organizational settings is discussed, including its potential role in making complexity-informed health promotion practical for all knowledge users.

## Introduction

Internationally, mental illness continues to represent a significant burden of disease, with this relative burden remaining largely unchanged since the 1990s (1). Although the COVID-19 pandemic has exacerbated this overall problem (1), it has also led to innovations in both prevention and treatment programmes and highlighted the key role of flexible and locally contextualized interventions and responses (2). The prevention literature identifies the importance of promoting community wellbeing and interventions to minimize developmental trajectories to formal mental health diagnosis (3). This scholarship highlights the inter-relationship between mental health, wellbeing, resilience, and protective and risk factors in both preventing and treating mental illness and promoting wellbeing and recovery. This article defines “mental health promotion activities” as intentionally delivered interventions and programmes designed to improve mental health (e.g., reducing anxiety or depression) and wellbeing outcomes (increases in thriving, flourishing, or mental wellness) at both the individual and collective levels. This definition values the interdependent nature of mental illness and wellbeing (4, 5). Governments, non-government agencies, programmes, schools, regions, and community networks have key roles in their design and implementation.

Improving mental health outcomes represents a “wicked problem,” given there are multi-factorial antecedents and tension points (6). To illustrate, there is a constant tension between biomedical (e.g., individual behavior change) vs. social determinants of mental health, with governments and policymakers often prioritizing one over the other (7). There are a range of additional tension points that bring complexity to mental health promotion. These include whether there is a design focus on: (i) mental health vs. wellbeing, (ii) individual vs. collective outcomes, and (iii) standardized vs. contextualized mental health delivery. Together, there has been an increasing call for “complexity” (or complexity science) to be the design and implementation principles for mental health promotion. This has been titled a move toward “complexity-informed health promotion” (8–11).

We argue that complexity must be embraced by policymakers, programmers, leaders, and communities within the design and implementation of community-based mental health and wellbeing interventions. In response, this article introduces the construct of “wellbeing-responsive community”: a vision and hierarchy of outcomes (or growth target) for intentionally delivered mental health promotion that embraces complexity as a design and implementation principle. This represents a community (team, agency, programme, region, school) that has the knowledge, language, methods, and skills to work side-by-side together to integrate best-practice science with their local knowledge systems and existing strengths and intentionally co-create contextualized wellbeing solutions at both the individual and community levels. The construct guides the design and implementation of interventions that respond to complexity and contextualization through the drivers of co-creation and local empowerment, which are made practical at multiple levels. Co-creation, as supported through the lens of transdisciplinary approaches, is emerging as an evidence-based method to respond to complexity (11, 12).

This article directly responds to the increasing call for complexity to be integrated within mental health promotion and the need for innovative tools, methods, and theories to drive this endeavor (8–10), as underpinned by collaborative approaches (13, 14). The construct extends the complexity-informed health promotion literature by drawing upon the language, approach, and set of methods of intentional practice (15) and understanding health promotion as a nested set of interventions spanning the “system” (e.g., whole-of-community) to the “moment” (e.g., intentional support, caregiving, or teaching). Traditionally, the mental health promotion literature does not routinely draw upon this “system” to the “moment” understanding and does not seek to isolate the common language and methods to empower all knowledge users in mental health promotion design and implementation (across all levels). Intentional practice, as a key pillar of the construct of a wellbeing-responsive community, affords the opportunity for communities to have a shared language and methods (founded upon intent) to design and implement interventions at both the individual and collective levels. It does so in a manner that embraces both complexity and contextualization. In other words, the construct may offer utility to make complexity-informed health promotion practical for all knowledge users (e.g., consumers, caregivers, teachers, principals, practitioners, clinicians, policymakers, and researchers). Traditionally, health promotion models have not sought to empower non-scientific knowledge users (e.g., consumers, caregivers, and teachers) in content related to complexity and contextualization. Our hope is that this article will inspire new insights into the intentional design and implementation of mental health promotion that empowers transdisciplinary knowledge users to embrace both complexity and co-construction. Resultantly, this leads to the spawning of locally contextualized interventions that span the “system” to the “moment”, promote individual and collective wellbeing, and reduce the burden of mental illness in the community.

## Underpinning design and implementation principle: embracing complexity

Drawing upon a programmatic approach (15), mental health promotion can be described as planned and intentionally delivered activities or methods that are linked through evidence to deliver a set of desired individual and collective outcomes. This programmatic definition, as summarized in Table 1, identifies three key features of mental health promotion: (i) outcomes, (ii) methods, and (iii) evidence.

**Table 1:** Programmatic features of mental health promotion.

**Key feature**	**Definition**	**Examples**
Outcomes	The desired target, growth impact, or goals of the mental health promotion activity or intervention.	• Increased wellbeing literacy • Building individual and/or community wellbeing • Increased resilience and mental wellness (e.g., reduced stress) • Reduction in mental illness symptomatology (e.g., anxiety and depression)
Methods	The activities, programme components, strategies, responses, and interventions.	• Social-emotional learning programmes • Community activities or events • Awareness raising programmes • Wellbeing and resilience skills training • Interventions addressing the social determinants of mental health
Evidence	The scholarly, theoretical, and local knowledge systems that support the logic between the desired “outcomes” and the “methods” to achieve them.	• Participatory evidence drawn from local knowledge systems • Contextualized logic model (and theory of change) • Empirical reviews • Evaluations and research

Table 1 summarizes the heterogeneous ways each feature can be made practical across real-world settings. This reflects the complex nature of mental health and wellbeing, and the diverse contexts in which mental health promotion is applied (e.g., across schools, agencies, regions, and communities). Traditionally, health promotion has brought a design focus to individualistic outcomes and singular or linear methods to achieve them (16).

### Complexity science and mental health promotion

There has been an increasing call for “complexity” (or complexity science) to be a design and implementation consideration for health promotion (8–11). This has been replicated across the broader mental health (17, 18), psychology (19), and positive psychology (20, 21) literatures. Complexity-informed health promotion understands that “health can be defined as an emerging complex product of the systemic interplay of many continuously co-changing ‘bio-cognitive-social-techno-environmental factors”' (8). In other words, drawing upon Table 1, complexity-informed mental health promotion can be operationalized as multi-leveled: (i) outcomes, (ii) methods, and (iii) evidence sources. This is further supported by intersectoral and multisectoral actions toward health [see review by Heard et al. (14)] or collaborative processes that span multiple stakeholders, government agencies, programmes, and non-government organizations (13). Although the evidence supporting multisectoral approaches is strong, there continues to be a lag in the implementation and best-practice mechanisms for this to occur (13, 14).

The following sections summarize four key domains of complexity (or tension points) that underpinned the conceptualization of a “wellbeing-responsive community”. We suggest that they are foundational design and implementation considerations for leaders, policymakers, and programmers to bring to all mental health promotion. They are outlined based on their respective continuums or tension points: (i) mental health vs. wellbeing, (ii) individual vs. community wellbeing, (iii) psychological vs. sociological determinants, and (iv) standardized vs. contextualized health design.

### Mental health vs. wellbeing outcomes

This article defines mental health promotion as interventions that improve mental health (e.g., reduce anxiety or depression) and wellbeing outcomes (e.g., increase thriving, flourishing, or mental wellness). However, the interface between mental health and wellbeing warrants attention. This relationship can be understood from two main perspectives: the dual vs. single continua models.

The single continua model views mental wellbeing as integral to mental health. It places mental health and wellbeing on a single spectrum, with mental illness/low wellbeing at one extreme and mental wellness/high wellbeing at the other (22). According to this model, mental health and wellbeing are distributed continuously in populations, and it is also possible to move in and out of those states. This approach draws heavily on a disease or pathogenic understanding of mental health, where there is an explicit assumption that one is either mentally unwell or mentally well. This “illness” vs. “wellness” viewpoint remains highly influential across psychiatry, clinical psychology, the medical literature, and across many service delivery organizations. Mental health promotion that draws upon a single continua model is likely to make generalized statements that improvements in wellbeing (or mental wellness, thriving, flourishing states) will have causal or direct improvements in reducing community mental illness.

In contrast, the dual continua model views mental health and wellbeing as interdependent but separate constructs (5). It proposes that an individual can have a mental illness and experience either flourishing (high mental wellbeing) or languishing (low mental wellbeing) states. In practice, this means a person can meet the diagnostic threshold for mental illness but also experience high levels of mental wellbeing. Similarly, a person can experience low levels of mental wellness but not meet the threshold for a mental health diagnosis.

We hold the view that mental health promotion needs to value the interdependent nature of mental illness and wellbeing as aligned with the dual continua approach (4, 5). This approach is embedded in widely accepted definitions of mental health and wellbeing (23). Drawing on the programmatic features outlined in Table 1, this means that mental health promotion should name their specific “outcomes” (e.g., reduced mental health symptoms and improved mental wellbeing) and identify the specific evidence-based strategies or “methods” that align with that stated outcome. We argue against programmers and policymakers making generalizations between mental health and wellness outcomes.

### Individual vs. community wellbeing

Wellbeing is a socially constructed term, embedded within cultural assumptions and values, and strongly influenced by liberal individualism [for review, see Christopher (24)]. Wellbeing has tended to be characterized via the individualist constructions of subjective wellbeing and satisfaction with life (25, 26), which draw upon hedonic, eudemonic (meaning), relational, and community engagement qualities [e.g., see PERMA; (27)]. It is routinely described in terms such as flourishing (27) and thriving (28, 29). Mental health promotion that has the intent to strengthen wellbeing would draw upon methods such as social-emotional skills training (including cognitive-behavioral therapy), awareness training, mindfulness programmes, workplace health promotion, and school-focused wellbeing curriculum. This is often linked to intermediate health promotion outcomes such as enhanced awareness and skill expression, and attitudinal or mindset reframing. In recent decades, the understanding and strengthening of wellbeing has been progressed through the positive psychology literature. This represents a strength-focused approach to human functioning (30), which is founded upon individualism (31).

Despite the increasing influence of positive psychology, there have been calls within the discipline to understand human functioning through the lens of systems (20) and the embracement of complexity science (21). Such approaches bring attention to the complex interdependence between individual and collective wellbeing (or community wellbeing). In alignment with this systems view, this article argues that community wellbeing is best understood as an interdependent or symbiotic relationship between the community and individual members (32). Lee and Kim (32) conceptualize community wellbeing as a collective process where community wellbeing is more than the sum of individual wellbeing. This brings alignment with the sociological construct of Gemeinschaft, which values the key role of social bonds within human functioning. Both system sciences and contemporary models of community wellbeing uphold the importance of sociological determinants to understand human and community functioning (20, 33). The concept of community wellbeing is of particular importance to marginalized and disempowered community groups. Inherent within the concept is a rights-based agenda. This recognizes the right of all people to equality of opportunity, access to services and supports, and to participate in the design and implementation of the services and programmes that impact their lives (34). To summarize, in reference to Table 1, we argue that mental health promotion needs to consider “outcomes” and “methods” that bring focus to both individual and community wellbeing.

### Psychological vs. sociological understandings

We hold the view that mental health promotion needs to uplift and value both psychological and sociological scholarships. In other words, wellbeing and mental health outcomes are best understood as the interface of biological, environmental, sociocultural, and psychological processes (35), or environmental vs. individual-level factors (36). This includes individual factors such as subjective wellbeing (25, 37, 38) and an individual's access to and engagement with environmental context; for example, community, relationships, green space, and health (39, 40). Within the developmental psychology literature, this is representative of the ecological model (41) or understanding “person-in-context” (29, 42). This seeks to understand the interface of both proximal (e.g., attitudes, values, skills, awareness, and biological) and distal factors (e.g., social support, access to employment and greenspace, and financial resources) to predict human functioning. There are literature examples of integrative models of mental health disorders that draw upon both psychological and sociological determinants (43).

Mental health promotion activities that are founded upon individualistic (or proximal) paradigms of human functioning will often focus on “outcomes” linked to knowledge, mental attitudes, and behaviors, rather than including broader structural activities that impact more directly on wellbeing. This redirection of attention to the inner self rather than the external social context may also be associated with a redirection of both private and public resources (33). This is reflected in the local South Australian research context, where there has been an incremental reduction in systematic health promotion activities that seek to address social determinants of health, with a stronger focus on biomedical or individualistic behavior change (7).

There is evidence that structural determinants, or sociological intervention points, have a stronger predictive impact on future health and wellbeing outcomes compared to psychological factors (23, 44, 45). The social determinants of health include the conditions in which people are born, grow, and live and which are shaped by the distribution of money, power, and resources at local and global levels. These have a key role in understanding and explaining individual functioning.

To illustrate, the provision of safe housing for a person experiencing homelessness is likely to achieve more effective wellbeing and mental health outcomes in contrast to a skill-focused psychological intervention (e.g., cognitive-behavioral therapy). From a political perspective, action on the social determinants of health is generally less palatable than instituting a lifestyle advice programme. The focus on the behavior of individuals is entirely consistent with political systems that use neo-liberal political philosophies that draw heavily on discourses of individualism (46). Such approaches focus on personal responsibility rather than broader government programmes and policies, which remain highly influential in government-based health promotion (47).

To summarize, we argue that the “outcomes” of mental health promotion must consider both psychological and sociological factors. In addition to individualistic outcomes such as reducing mental health symptoms or improving wellbeing, broader community and sociological outcomes are also brought into focus. This may include factors such as group belonging and solidarity, school connectedness, community participation and engagement, access to greenspace or medical support, strengthening education, housing, income security, employment, transport for disadvantaged communities, and broader community resources.

### Standardized vs. contextualized health promotion delivery

The implementation of mental health promotion can differ markedly in the degree to which they are standardized rather than contextualized to individual/community needs and context. A strong argument is made in the literature for the role of standardized approaches, which draw upon prescriptive guidelines articulating the relationship between “outcomes,” “methods,” and their supporting “evidence”. Meta-analyses consistently show that programme fidelity, or consistency of delivery as per the intended design, remains a strong predictor of intervention outcomes (48, 49).

Despite this, the effectiveness of mental health promotion is also mediated by a range of contextual factors such as age, gender, culture, trauma background, historical factors, and personality (48–51). This requires attention to be paid to “person-activity fit” (52) and uplifting the principles of contextualization and personalization to intervention design, adaptation, and implementation (53). Contextualized interventions blend theories and best-practice evidence in a manner that is responsive to local conditions and context (54) and seek to find the “right mix of fidelity and adaptation” (48) or “flexibility within fidelity” (55). The importance of contextualization is particularly highlighted for child and youth cohorts, given their unique and changing developmental needs (56). In summary, we hold the view that mental health promotion needs to value contextualization (or personalization) in its design and implementation. This should occur in a manner that balances flexibility and fidelity and is informed by principles of evidence-based adaptation (57).

### Summarizing complexity

The previous sections have highlighted four continuums of complexity, or tension points, for policymakers, programmers, and communities to consider as key design and implementation principles within mental health promotion. If complexity is not adequately understood nor defined sufficiently, then there is a high risk that interventions will be poorly executed or translated, and finite public and private resources will be applied in inefficient ways. However, embracing complexity within mental health promotion is complex in its own right and not an easy task. There is a need for innovative tools, methods, and theories to advance this endeavor (8–10), as supported by collaborative approaches (12–14). It is from this complex context that the construct of “wellbeing-responsive community” was conceptualized.

## Wellbeing-responsive community

### Participatory development

The conceptual development of a “wellbeing-responsive community” emerged through a 5-year participatory process involving industry collaborations across education, community services, mental health, and healthcare across Australia. Its development was motivated by the call to embrace complexity in the design and implementation of mental health promotion and the importance of transdisciplinary approaches that value all knowledge users in the process of knowledge production and intervention design and planning (12). It has been inspired by the knowledge translation literature (58) and the deconstruction of scientific evidence for non-scientific audiences (59). For this reason, the concept of a wellbeing-responsive community is framed and defined by applying non-scientific (or inclusive) language to offer a common language bridge for scientific and non-scientific knowledge users to design, adapt, and implement mental health promotion activities. It draws upon the importance of individuals and communities having shared “mental models” of the intent (or outcomes) of their collaboration or health promotion activities, thereby strengthening implementation quality (60).

### What is a wellbeing-responsive community?

**Figure 1:**
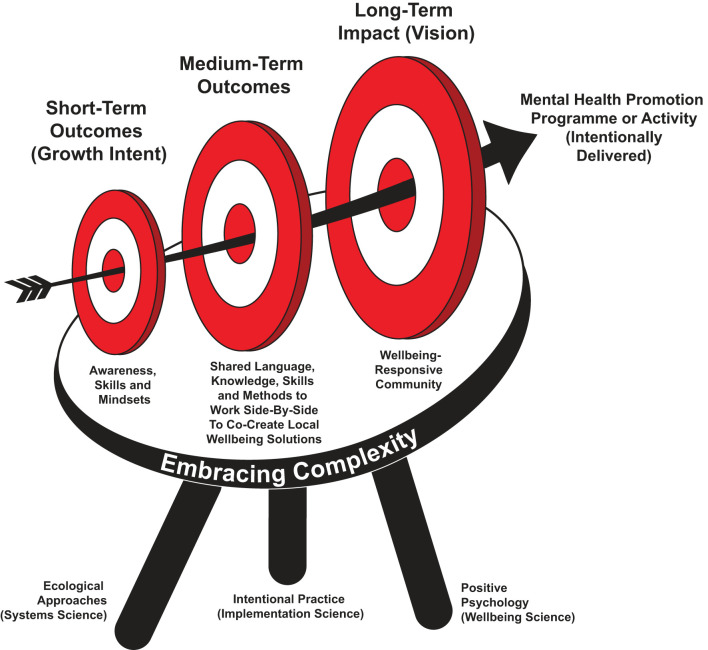
Wellbeing-responsive community: growth target for mental health promotion.

Figure 1 graphically represents a wellbeing-responsive community as a growth target or set of intended outcomes for intentionally delivered mental health promotion. In short, drawing upon the programmatic features outlined in Table 1, the construct is solely focused on identifying a cluster of “outcomes” that underpin the design principle of embracing complexity. By definition, the long-term outcome, impact, or vision of a wellbeing-responsive community is as follows:

“*a* community *which is* responsive *to the* wellbeing *and* growth needs *of individual and collective community members, translating to a* connected thriving local community”.

Each of these underlined terms is defined in Table 2.

**Table 2:** Definition of wellbeing-responsive community.

**Feature**	**Definition**
Community	A group of people who are brought together around a shared purpose. It may include a support team, school, programme, government department, network, or region.
Responsive	The community has an awareness of the needs and context of individual and collective community members and has the language, knowledge, methods, and skills to work side-by-side together and intentionally respond to these needs in a contextualized or personalized manner.
Wellbeing	A mental health and wellbeing outcome which is operationalized in the language of the community and reflects the interdependence between (i) mental health and wellbeing (dual continua) and (ii) individual and community wellbeing.
Growth needs	The wellbeing, trauma, developmental, therapeutic, medical, economic, social-cultural, and life needs of individual and collective community members.
Connected thriving local community	A community where human connections and shared solutions are evident; individual and collective growth needs are met; and meaning, aspiration and satisfaction are expressed at the individual and collective levels.

### Epistemological foundations: embracing complexity and systems informed positive psychology

As graphically represented by Figure 1, the wellbeing-responsive community is founded upon the embracement of complexity (see previous sections). It aligns with the epistemological, political, and ethical assumptions of Systems Informed Positive Psychology [SIPP; (20)]. At the heart of both SIPP and wellbeing-responsive community is “interdependency” or the co-existent or symbiotic relationship between humans, community, environment, and wider systems.

### Three scientific pillars

The definition, categorization, and applied application of wellbeing-responsive communities are drawn from three scientific pillars: (i) positive psychology, (ii) ecological or systems approaches, and (iii) intentional practice (implementation science). These are depicted by the three supporting legs of the growth target (see Figure 1).

#### Positive psychology

Positive psychology is an “umbrella term” (61) capturing a broad stream of theories and applications focused on strengthening human wellbeing and wellness (30). It draws upon an applied approach to optimal functioning and considers the strengths, virtues, and processes that enable individuals, communities, and organizations to thrive or experience optimal wellbeing (61). It has developed from a rebuke of pathogenesis, an illness or deficit orientation to understanding psychological functioning, or a preoccupation with psychological problems (e.g., stress, clinical symptoms, anger, aggression, and negative personality traits) that has been dominant across psychology (62).

Wellbeing is a fundamental construct within positive psychology (25). “Positive psychology interventions” (PPIs) have emerged as empirically tested strategies, exercises, and activities designed to promote happiness and wellbeing (63). PPIs within the literature focus on outcomes such as optimism, meaningfulness, resilience, gratitude, kindness, and compassion. PPIs draw upon a range of strategies, activities, and methods, including character strength identification, mindful awareness, savoring approaches, goal setting, and coaching techniques.

Wellbeing-responsive community as a construct is grounded in the positive psychology literature in the following ways:

- It brings a strength-focused orientation to mental health promotion and encourages a move away from theories or approaches founded upon a disease- or deficit-based model.
- It understands that there is an important role for both wellbeing and proximal factors (e.g., knowledge, values, attitudes, skills, mindsets, and beliefs) in understanding human functioning.
- It supports the key role of intentionally delivered and evidence-based interventions (e.g., PPIs) in mental health promotion.

#### Ecological or systems science

Previous sections have highlighted the role of psychological and sociological understandings to be integrated within mental health promotion. In other words, wellbeing and mental health outcomes are best understood as the interface of biological, environmental, sociocultural, and psychological processes (35). This is captured through the ecological systems model (41, 42, 64, 65) which sees a “reciprocal and transactional” relationship between a person and their societal context [(66), p. 432]. According to Bronfenbrenner, human development is influenced by five nested levels: the microsystem (e.g., home environment), mesosystem (e.g., school), ecosystem (e.g., the environment supporting teachers), macrosystem (e.g., cultural values), and chronosystem (e.g., major life events and COVID-19). Each of these systems interacts with and influences each other to explain human functioning.

This systems approach asks organizations, schools, and communities to move away from a “reduce and resolve” approach to health promotion to one that values a dynamic worldview that supports self-organization and adaption (67). This involves participants and knowledge users working together in a dynamic and flexible approach to planning and implementation that is founded upon local empowerment and shared exploration (68).

The construction of a wellbeing-responsive community is strongly informed by ecological system sciences in the following ways:

- It explicitly understands that every individual and community has a unique context and that mental health promotion needs to be contextualized (or personalized) to both individual and collective needs and context.
- Context can be understood as the interaction of multiple levels or systems, including home environment, the community setting (mesosystem), community values and attitudes, and broader societal events and changes.
- The target or desired outcomes of mental health promotion should consider both proximal (e.g., values, attitudes, skills, and mindsets) and distal factors (e.g., contexts/systems).
- Through local empowerment, communities or groups have the capacity to dynamically self-organize and develop their own solutions.

#### Intentional practice (implementation science)

The third underpinning pillar of a wellbeing-responsive community is intentional practice. This is a common language, approach, and set of methods, empirically located within the implementation science literature, that supports both scientific and non-scientific knowledge users to design, adapt, and implement contextualized “wellbeing solutions” (53). Raymond (53) defines wellbeing solutions as “any strategy, intervention, program or response that is designed to deliver a wellbeing, growth, learning, developmental, behavioral or therapeutic outcome” (p. 2). By this definition, individual and collective mental health promotion activities represent wellbeing solutions.

Intentional practice offers a common language, approach, and set of methods to operationalize wellbeing solutions spanning the “system” (e.g., community level) to the “moment” (e.g., intentional support). This includes “whole-of-community” capacity-building initiatives, wellbeing and resilience skill-building interventions, and moment-to-moment support that responds to an individual's needs and context (e.g., trauma-informed practice). Importantly, intentional practice holds the view that intentionally delivered interactions and support occurring between two or more people are meaningful “wellbeing solutions” in their own right. This offers a more nuanced understanding of wellbeing and mental health interventions than is traditionally seen in the literature. It means that mental health promotion can be characterized as a myriad of nested or multi-layered contextualized interventions or wellbeing solutions.

Intentional practice is defined as both an “approach” and “set of methods” (53). As an approach, it asks scientific and non-scientific knowledge users to hold onto principles of complexity and contextuality and bring a lens of mindful awareness, growth, and intentionality to the design and implementation of wellbeing solutions. As a set of methods, it offers models, critical questions, and process-based tools to bring together best-practice evidence with local knowledge systems, as supported through co-creation. Intentional practice is founded upon the proposition that mental health promotion activities founded upon the principles of awareness, growth, and intentionality (and drawing upon key intentional practice methods) are more likely to deliver stronger outcomes and reduce unintentional harm or negative consequences.

In its most practical application, intentional practice asks knowledge users to bring ongoing awareness to the intent or purpose of their mental health promotion activity. Drawing upon Table 1, this includes awareness of both the “outcomes” (or “what”) and “methods” (or “how”) in design, adaptation, and implementation (53, 69, 70). A feature of intentional practice is that it offers a shared language and approach for scientific and non-scientific knowledge users. It argues that complexity in mental health and wellbeing can only be adequately understood and responded to through people and communities coming together in “shared intent” in intervention design, adaptation, and implementation (53). Raymond defines this as a community or group having a “shared and co-created awareness of (1) what is happening in the wellbeing solution context, (2) what is the intent or desired outcomes of the wellbeing solution, and (3) how this will be collectively actioned” (p. 6). Intentional practice is purported to offer significant utility across educational, mental health, trauma-informed, complex programming, culturally sensitive practices, and community mental health settings [for review, see Raymond (53)].

Wellbeing-responsive community draws upon intentional practice in the following ways:

- The immediate intent (or “growth intent”) of mental health promotion (and all its nested wellbeing solutions) should be clearly articulated and brought to ongoing awareness in design and implementation across all knowledge users and community members. This is representative of “intentional mental health promotion”.
- Across communities, there is a myriad of nested contextualized wellbeing solutions spanning the “system” to the “moment” (intentional support, caregiving, or teaching). They are delivered by scientific and non-scientific community members, including parents, families, teachers, practitioners, supporting adults, and programmers.
- There is a key role for a common language, approach, and set of methods to design, adapt, and implement contextualized wellbeing solutions across a community.
- It is only when community members come together through “shared intent”, founded upon common language and methods, where local knowledge systems are empowered, that complexity and contextualization can be adequately understood and addressed. In other words, communities can be empowered to learn the “process” of developing their wellbeing or health promotion solutions, founded upon evidence and local knowledge systems.

## Hierarchy of outcomes and applied definitions

Drawing upon these three scholarly areas, the construct of wellbeing-responsive community is exemplified through a hierarchy of short-, medium-, and long-term outcomes, represented by the three targets in Figure 1. These outcomes are defined in Tables 3, 4. The hierarchy of outcomes is categorized through the Life Buoyancy Model (LBM): a foundational intentional practice model or programme logic framework (69, 70).

**Table 3:** Wellbeing-responsive community: hierarchy of outcomes.

**Short term (growth intent)**	**Medium term**	**Impact (vision)**
**Awareness** • Wellbeing, mental health, resilience, growth, trauma, and optimal human development. • Proximal and distal factors that drive wellbeing, growth, and mental health outcomes for people with diverse contexts. • Self, actions, and impact of actions on others. **Skills** • To intentionally respond (rather than react) to others through moment-to-moment relationships, growth planning processes, programming, and intentional support processes. • To co-design wellbeing solutions for individual and collective community members. **Mindsets** • “I belong to a community where mental health and wellbeing is important and is valued.” • “I bring a growth intent to the wellbeing of myself, the people I support and my community.” • “I have the skills and confidence to work side-by-side with others and my community to improve wellbeing outcomes.”	Community has the capacity (through language, knowledge, methods, and skills) to: • Understand the science of wellbeing, trauma, growth, and optimal human development. • Integrate this knowledge with local wisdom and existing strengths. • Apply this knowledge intentionally. • Work side-by-side to co-create personalized individual and community wellbeing solutions (through shared intent).	A community which is responsive to the wellbeing and growth needs of individual and collective community members; translating to a connected thriving local community.

**Table 4:** Definitions of medium-term outcomes.

**Outcome**	**Definition**
1. Understand the science of wellbeing, trauma, growth, and optimal human development.	The community has foundational knowledge of widely recognized empirical evidence on the key principles, methods and strategies to build optimal mental health and wellbeing outcomes, as matched to individual and collective context (e.g., trauma, wellbeing, and systems science).
2. Integrate this knowledge with local wisdom and existing strengths.	The community can internalize and make sense of this understanding within their existing knowledge systems and local context.
3.Apply this knowledge intentionally.	The community is applying this understanding in an intentional manner through expressed actions, including moment-to-moment support to others, mental health and wellbeing programming, growth and care planning, community responses and interventions, and whole-of-community approaches or initiatives.
4.Work side-by-side to co-create personalized individual and community wellbeing solutions (through shared intent).	Community members can work side-by-side in “shared intent” to design and implement responses, interventions, programmes, and support which is tailored to the needs and context of individual and collective community members. This is founded upon intentional practice design and implementation principles (and methods) and drawing upon best-practice understandings of mental health and wellbeing and local knowledge systems.

In the short term, a wellbeing-responsive community asks programmers, policymakers, and practitioners to bring ongoing focus to the intent of their mental health promotion. Drawing upon the language and definitions of intentional practice (53), this represents the immediate “growth intent” of health promotion. As graphically represented in Figure 1 (growth target), they remain highly visible in the design, adaptation, and implementation of all mental health promotion activities. This set of short-term outcomes is logically linked to the medium- and long-term outcomes. They are categorized by applying the intentional practice descriptors of: (i) awareness (knowledge or insight), (ii) skills (expressed actions), and (iii) mindsets (attitudes or beliefs). They represent the core competencies held by a wellbeing-responsive community and the immediate growth target for intentionally delivered mental health promotion.

Through the acquisition of short-term growth, the medium-term outcome is that the community develops the shared language, knowledge, skills, and methods to come together and develop wellbeing solutions (interventions, programmes, or responses) that can directly respond to the unique needs and contexts of individual and collective community members. This occurs through the community having: (i) a shared understanding of best-practice knowledge, (ii) the ability to integrate this with their existing knowledge systems, (iii) the capacity to apply this knowledge through intentional actions, and (iv) the capacity to co-create shared wellbeing solutions, or contextualized interventions and responses, founded upon shared intent. Definitions of the medium-term outcomes are provided in Table 4.

Through the medium-term outcomes being achieved, the long-term outcome or vision of a wellbeing-responsive community is a community that is responsive to the wellbeing and growth needs of individual and collective community members, translating to a connected, thriving local community.

## Applications

The construct of a wellbeing-responsive community offers a cluster of “outcomes” embracing complexity that would appear to offer the most utility within “community capacity building” or “strengthening” health promotion initiatives (71, 72). In other words, to empower community action to improve local health and wellbeing outcomes (71).

As per its definition, “community” is not defined by geography nor location, but represents a group of people brought together through a shared intent or set of needs or goals. For this reason, it is postulated to offer utility across multiple communities, big and small. This includes a support team spanning 3–5 people, school, agency, network, or geographical area. The applied definitions of the outcome hierarchy (see Tables 3, 4) are not embedded within a specific discipline or setting. The construct is postulated to offer utility in bringing together transdisciplinary teams and both scientists and non-scientists alike. It offers both generalized and context-specific applications.

### Generalized applications

#### Mental health and wellbeing programme design, adaptation, and implementation

The concept of a wellbeing-responsive community can strengthen the design, adaptation, and implementation of a variety of mental health promotion activities. In its simplest form, the construct asks leaders, policymakers, programmers, and communities to clearly articulate the intent or purpose of their mental health promotion activity. It offers a hierarchy of outcomes (Table 3) that can be integrated into the design, adaptation, and implementation of existing or new health promotion activities, mental health strategies, or community capacity-building initiatives. The specific methods, strategies, and components to deliver these outcomes are co-designed with the local community. The construct supports the important role of intentional programme design and implementation, founded upon evidence (15, 53), and in a manner where there is a clear logic between the outcomes and methods to achieve them (see Table 1).

#### Uplifting shared process-based factors within mental health promotion

A wellbeing-responsive community offers an outcome hierarchy that illustrates the “process” of co-creating contextualized wellbeing solutions founded upon evidence and local knowledge systems. It seeks to empower communities to work side-by-side together in “shared intent.” This is a participatory process that sees community members as shared citizens and scientists in health promotion (73). Drawing upon intentional practice (53), wellbeing solutions or interventions can be applied at the individual or collective level, as well as through moment-to-moment support and system responses (e.g., from the “system” to the “moment”). In other words, it is focused on communities having the capacity to intentionally respond to the growth, developmental, or wellbeing needs of individuals and groups in a manner that draws upon ecological (psychological and sociological) understandings of mental health and wellbeing. Capacity is defined as community knowledge, skills, methods, and language. This offers an empowerment approach to mental health promotion, as opposed to being content-driven. The latter is representative of interventions and programmes that bring an intent to specific mental health and wellbeing outcomes (anxiety, depression, wellbeing, etc.). It draws upon the metaphor that a wellbeing-responsive community has the local capacity to “learn how to fish” or co-construct evidence-based wellbeing solutions matched to individual and collective context. This contrasts with a community being provided with an external programme that “offers them [or imposes] the fish.” In other words, they are provided with an external content-driven programme linked to specific mental health and wellbeing outcomes.

### Context-specific applications

The construct of a wellbeing-responsive community is postulated to have utility across the following applications.

#### Support teams (communities)

Across many mental health, wellbeing, and community service settings, teams of people are entrusted to work side-by-side with clients, consumers, and community groups to support shared growth outcomes. A wellbeing-responsive community raises the following question: “how might a support team be reframed as a support community”? The latter may offer a more inclusive and less hierarchical approach to shared support and care. The hierarchy of outcomes characterized by a wellbeing-responsive community can seek to guide the intent of a support community around a client or consumer. In other words, a support community's shared intent could be defined as to “be responsive to the client's wellbeing and growth needs, and we action this by working side-by-side with the client to co-design shared wellbeing and growth solutions”. The construct of a wellbeing-responsive community also identifies a range of outcomes (e.g., Table 3) a consultant, trainer, coach, or lead clinician could bring to their intent to build the capacity of the support community to deliver this outcome. It supports the evidence that transdisciplinary collaborations work best through common aims, methods, and working processes (74).

#### Education and schools

Schools and education systems are increasingly being asked to consider the wellbeing, developmental, growth, and mental health needs of students. This includes bringing increasing awareness to systems thinking and complexity science (67). Across school-based health promotion (or health-promoting schools), the literature identifies a range of challenges around implementation, with a focus on translation and integration of practices (75). The outcome of the wellbeing-responsive community can be applied as an overarching vision or outcome hierarchy for a school community. It offers a hierarchy of outcomes (Table 3) that can be integrated into the design and review of “whole-of-school” capacity-building initiatives (or wellbeing strategies). This offers a holistic approach to student and community wellbeing that moves away from individualistic and reductionist approaches.

It is not uncommon for schools to identify mental health promotion through compartmentalized features such as social-emotional learning packages, wellbeing curriculum (including positive psychology interventions), and specialist roles (wellbeing counselors, pastoral care workers). In contrast, a wellbeing-responsive community brings a holistic focus to student wellbeing where all community members (e.g., educators, students, leaders, and families) have a role in building student wellbeing, drawing upon both psychological and sociological determinants. It is focused on developing the shared language, skills, methods, and knowledge within a school community. This means that educators, administration staff, leaders, families, and students are empowered to work side-by-side together in shared intent around locally owned wellbeing, mental health, and growth solutions. A further feature of the construct is that it moves the thinking and planning of mental health and wellbeing beyond the geographical boundaries of the school and asks the question, “how can our school be part of a broader wellbeing-responsive community”?

#### Community groups, agencies, programmes, and networks

There are a range of government agencies, non-government sites, and programmes that are dedicated to supporting others to build wellbeing, mental health, recovery, and whole-of-life outcomes. These groups often deliver a range of interventions, responses, and programmes. It is not uncommon for these interventions and responses to occur in isolation or in a compartmentalized manner. The construct of a wellbeing-responsive community can offer a group, agency, programme, department, or region an overarching set of outcomes that serve as an organizing framework for collective action or shared intent for both existing and new initiatives. It asks communities and groups the following key questions:

- Do we have the shared language, knowledge, skills, and methods to develop shared wellbeing or growth solutions that respond to the needs and context of individual and collective community members?
- Are we intentionally delivering our existing programmes and services in a manner that is matched to the needs and contexts of individual community members?
- What is the intent of our existing programmes and interventions, and how does this integrate with other programmes and interventions to build a wellbeing-responsive local community?

We suggest that an important application of wellbeing-responsive community is the design and implementation of mental health promotion across marginalized communities. These are communities that include members with diverse needs and presenting behaviors (e.g., criminal behavior, drug and alcohol use, and homelessness), which require a nuanced response. Traditional health promotion models and approaches are not routinely effective with marginalized cohorts (76). A wellbeing-responsive community offers a hierarchy of outcomes that is designed to empower both consumers and health practitioners to come together in shared intent and develop locally owned solutions (or mental health promotion activities) at the individual and collective level. In other words, it offers a set of shared competencies for a community to come together and construct their own solutions. Mental health promotion is not done “on” or “to” a community, but instead is co-constructed or done “with” the community.

## Discussion and future directions

Improving community mental health outcomes represents a “wicked problem” (6). We have highlighted the key areas of complexity and tension points that leaders, programmers, policymakers, principals, and researchers should consider within the design and implementation of mental health promotion. This aligns with the literature move toward “complexity-informed health promotion” (8–11). This article has responded to the need to develop innovative tools, methods, and theories to characterize and apply this endeavor (8–10), as supported by transdisciplinary approaches (12–14).

We have introduced the construct of a “wellbeing-responsive community”: a vision and hierarchy of outcomes (or growth target) for intentionally delivered mental health promotion that embraces complexity as a design and implementation principle. This represents a community (team, agency, programme, region, or school) that has the knowledge, language, methods, and skills to work together in shared intent, integrate best-practice science with their local knowledge systems and existing strengths, and intentionally co-create contextualized wellbeing solutions at both the individual and community levels. The construct offers a novel method to respond to complexity and contextualization through the drivers of co-creation and local empowerment, as defined at multiple levels. Co-creation, or transdisciplinary collaboration, is emerging as an evidence-based method to respond to complexity (11). The construct extends the complexity-informed health promotion literature through its integration of intentional practice (15), the first mental health promotion article to do so. Intentional practice offers the unique understanding that mental health promotion can be exemplified as a myriad of contextualized wellbeing solutions (interventions) that are nested within each other and span the “system” (e.g., whole-of-community) to the “moment” (e.g., intentional support, caregiving, or teaching). Traditionally, the mental health promotion literature does not routinely draw upon this “system” to the “moment” understanding, nor seeks to isolate the common language and methods to empower all knowledge users in mental health promotion design and implementation (across all levels). Given the purported benefits of communities having a shared language and methods (founded upon intent) to design and implement contextualized interventions, the construct of a wellbeing-responsive community may offer utility to make complexity-informed health promotion practical for all knowledge users (e.g., consumers, caregivers, teachers, principals, practitioners, clinicians, policymakers, and researchers).

This article offers a contextual, scholarly, and applied case for introducing the construct of wellbeing-responsive community as a growth target (or outcome hierarchy) for community capacity-building or strengthening initiatives. Although we offer a range of purported applications for the construct, we draw no conclusions regarding its relative utility nor translatability. We suggest the construct is uniquely placed to inform the design and implementation of mental health promotion across marginalized communities, where there are questions regarding the utility of traditional health promotion models and approaches (76). We note that further empirical and applied work is required, with a focus on the following five key areas:

- *Integration within transdisciplinary programme design*—to assess its utility across transdisciplinary settings, the hierarchy of outcomes should be integrated into logic models that are co-designed with individual schools, agencies, teams, and communities.
- *Development of tools, programmes, and methods that are mapped to the hierarchy of outcomes*—there is a need for evidence-based and validated tools, strategies, and intervention components that are mapped against the hierarchy of outcomes. This is best served through action-based research processes. This work is already underway and will be the focus of future publication.
- *Participatory feedback*—the utility of a wellbeing-responsive community as a shared community narrative and set of outcomes warrants further assessment and participatory review. The concept has been designed to be owned, understood, and applied by diverse communities, and as such, there is a need to assess the degree to which this can occur.
- *Measurement tools*—there is a need to develop operational definitions to ensure consistency in measurements of the hierarchy of outcomes proposed in this article and to identify associated assessment tools and processes. These should then be integrated into the monitoring and evaluation of programmes and responses that apply a wellbeing-responsive community as a set of outcomes.
- *Mixed method evaluation*—community-based programmes and interventions drawing upon the wellbeing-responsive community hierarchy of outcomes should undergo mixed method evaluation, with attention paid to the predictive relationship between the short-, medium-, and long-term outcomes (Table 3).

## Summary

Strengthening and improving community-based wellbeing and mental health outcomes continues to be a key public health focus. This article highlights the importance of embracing complexity in the design, review, and implementation of mental health promotion. A wellbeing-responsive community is offered as a novel construct and outcome hierarchy (or growth target) to support the design and implementation of intentional mental health promotion. It has been designed to honor and capture complexity and draw upon the features of contextualization, co-construction, local empowerment, and intentionality. By uplifting the “processes” of designing and implementing locally contextualized solutions, it is postulated to offer a transdisciplinary and unifying construct for communities to deliver local mental health and wellbeing outcomes. It is founded upon the premise that the burden of mental illness within our community can only be reduced when communities, big and small, come together in shared intent. In other words, they are empowered to embrace complexity in the design and implementation of locally owned and delivered solutions.

## Data availability statement

The original contributions presented in the study are included in the article/supplementary material, further inquiries can be directed to the corresponding author.

## Author contributions

IR: Conceptualization, Writing—original draft, Writing—review & editing. KB: Conceptualization, Writing—original draft, Writing—review & editing. KA: Conceptualization, Writing—original draft, Writing—review & editing. DK: Conceptualization, Writing—original draft, Writing—review & editing.
